# Conceptualizing Context and Intervention as a System in Implementation Science: Learning From Complexity Theory: Comment on "Stakeholder Perspectives of Attributes and Features of Context Relevant to Knowledge Translation in Health Settings: A Multi-country Analysis"

**DOI:** 10.34172/ijhpm.2021.147

**Published:** 2021-10-25

**Authors:** Lisa M. Pfadenhauer

**Affiliations:** ^1^Institute for Medical Information Processing, Biometry and Epidemiology - IBE, Ludwig Maximilian University of Munich, Munich, Germany.; ^2^Pettenkofer School of Public Health, Munich, Germany.

**Keywords:** Context, Implementation Science, Complexity Theory

## Abstract

In implementation science, implementation has been widely theorized and assessed. Context, on the other hand, usually played a minor role in the field and was usually conceptualized in a rather positivist way. Despite some promising efforts, there is a strong need to continue building theory on context and operationalizing the concept in implementation practice. I argue for the benefit of integrating complexity theory into our understanding of context in order to further our thinking about context and intervention as a system. This should be reflected by the way in which we build theory as well as apply this theory by employing methods that adequately account for complexity in systems.

## Introduction

 Over the past decades, implementation and knowledge translation have been widely conceptualized and assessed. While there is general agreement as to what constitutes implementation, the definition of context has less consensus. Indeed, context is usually defined only indirectly by its determinants (eg, patients; organizational culture and climate; organizational readiness to change; organizational structures; organizational support; social relations and support, financial resources, leadership, time availability, feedback and physical environment^1^) or by what it is not (ie, everything external to the intervention itself).^1-4^ This partial maturity of the concept has been subject to several attempts to advance it and move the field towards employing a common understanding.^2,3^

 In both implementation science theory and practice, however, context remains a contested concept. This not only hinders us from furthering our understanding of context in implementation science and building adequate theory, but also prevents us from operationalizing it in implementation practice.

 I argue how the rather positivist thinking in implementation science theory and practice can benefit from complexity theory and advocate for the application of a plurality of methods that better attend to the principles of complex systems thinking.

## Integrating Complexity Theory Into Implementation Science

 In order to understand why context in implementation science has been conceptualized in this way we need to look at the influential paradigms in implementation science.

 Implementation science as an offspring of the evidence-based medicine movement has largely grown and developed under a *positivist* paradigm. Stripping away context by designing, implementing and evaluating (clinical) measures under controlled circumstances is just one of the inherent traits of the evidence-based medicine movement, a trait which has been taken forward to implementation science.^5^ This paradigm implies that there is one reality by studying the respective parts of a system (intervention, implementation, context, outcomes) as distinct components. The link between these parts was assumed to be rather straightforward.^6^ Subsequently, contextual aspects have been – and are – assessed in implementation science as individual factors that act as barriers or facilitators to interventions that can be overcome or utilized by implementation strategies. This view of context involves examining individual factors within a specific context as potential confounders or moderators which add up to a whole and predictably lead to a certain and inherent effect. By assessing the parts of the system, the whole can be understood. Some of the criticism imposed on this positivism has been responded to by *post-positivism,* which acknowledges that the link between a cause and effect is contextually bound and thus generalizing becomes difficult.^7^

 Of lesser importance in implementation science have been paradigms such as *complexity theory*. Complexity theory offers the concepts of complex systems and system thinking.^8^ A system has been defined as a set of interconnected elements, whose interactions form a whole to produce a coherent set of functions.^9^ Interventions are considered events in these systems.^9^ Interactions and feedback loops between individual actors and artefacts evolve in unpredictable, uncertain and non-linear ways, leading to both intended and unintended consequences. Adaptive systems have fuzzy boundaries, are highly context dependent in terms of time, history, and space, including location and proximity.^10^ Most importantly, complexity theory requires the researcher to keep the whole system in mind when assessing its parts.^9^ Thus, it looks at what the system *does* rather than what it *is.*^5^ Where applied in implementation science, complexity theory offers valuable insights: triggers for stimulating change, necessity of feedback loops, time, as well as the added value of conceptualization of context as complex system to further our understanding of these processes.^6^

## Employing an Organic View on the Intervention – Context Dyad

 Developing or adapting and implementing a (complex) intervention cannot happen without changing the way we conceptualize context. Following complexity theory, this implies to conceptualize intervention and context as a dyad, a co-evolving organism. Interventions, measures and programs are embedded into a specific context – either by researchers or other agents – and will become part of this context. This applies both to newly developed as well as evidence-informed interventions. The recently published ADAPT guidance^9^ which provides guidance on how to intentionally modify evidence-informed interventions in order to achieve a better fit with a new context follows this train of thought. It postulates moving from conceptualising implementation as a process of implanting evidence-based interventions into organisms with high fidelity towards conceptualising it as a reciprocal process where both the intervention and the context undergo adaptation. Speaking in realist terms, we will have to envision an intervention as re-configuration of the context. This will imply a shift away from the question whether or not an intervention fixes a problem but rather, if it reshapes the system in favourable ways.^11^ Currently, this is not sufficiently captured in frameworks, models and theories (FMTs) in implementation science, where context still constitutes one of many components rather than a core aspect.^1^ While this has consequences for the design of an intervention, it has also consequences for how we assess context: context should not be described but analysed with regards to the caused leading to certain outcomes within a respective context. Most importantly, this organic view of the intervention-context dyad sensitizes the researcher to intended and unintended effects.

## Adapting Framework, Model and Theory Building

 Naturally, these paradigms are reflected in the way the field of implementation science constructs its FMTs. With positivism exerting a strong influence in implementation science, it comes as no surprise that methods such as evidence syntheses and systematic reviews have been repurposed in order to build theory. It is, thus, not surprising that many of the FMTs commonly cited are deduced from previous FMTs,^4^ forming, to a varying extent, meta-theories.^12^ It is therefore also not surprising that there is significant overlap between FMTs and their modifications.

 While this route could lead to more consolidated FMTs, they are still quite inconsistent when it comes to certain elements, such as place or characteristics of the individuals receiving an intervention. This indeed raises the concern about the comprehensiveness of any of these FMTs and lastly about the process of theory building in implementation science.^12^ Potentially, more inductive approaches exploring and understanding context could explore and elucidate these inconsistencies.

 Squires et al aim to address this by employing a rather inductive approach to explore perspectives on context in implementation by exploring subjective realities and views of context.^12^ They do so not by presenting an a-priori definition of context but allowing experts to elicit their understanding.^12^ Thus, they add an important first step to openly exploring the structures, patterns and characteristics that individuals ascribe to a respective context (ie, the physical place where an intervention is delivered) as well as context and how it evolves over time.

 One of the characteristics identified through this approach is the *facility or spatial context*.^12^ Indeed, a recent scoping review of determinant frameworks used in implementation science revealed that the least common dimension addressed by included frameworks was the physical environment.^1^ While spatial aspects such as location and proximity emerged as critical in public health informed by complexity theory,^2,10,13^ it has been less frequently taken up by determinant FMTs on implementation in healthcare.^1^ Considering the importance stakeholders impose on this aspect,^12^ this should, however, be taken up in implementation science FMTs.

 Interestingly, *time* seems to only be of importance when it comes to the lack of time available for the adoption of a new intervention.^12^ This aligns with other FMTs where time was attributed only a minor importance, and if considered, only related to a lack of resources in the people delivering and receiving an intervention.^1^ This seems rather surprising since failing to account for the dynamic and situational nature of adaptive systems might prevent intervention designers, implementers and evaluators from responding to emerging challenges and changes, and responding to them accordingly.^9^ Equally, the temporal dimension is of high interest for stakeholders when assessing and interpreting the effectiveness of interventions at a certain point in time.^9^ Timing – rather than time (or lack thereof)– indeed can be everything. For stakeholders acting and embedded within complex systems, recognizing and acknowledging the dynamic nature of a certain complex system may be challenging. Therefore, while inductive approaches such as interviews can reveal novel concepts in context, they might also fall short in capturing certain aspects. This points towards employing methodological pluralism in order to account for the many dimensions contributing to complexity in context.

 At the macro level (ie, external to the organizational meso context), the stakeholders consulted by Squires et al identified the political climate as well as regulatory and legislative standards.^12^ Thus, they acknowledged the influence of an otherwise underdeveloped and underassessed level of context in implementation science.^1^ While it will remain challenging or impossible to establish causality between factors on the macro level and outcomes on a meso or micro level,^1^ key aspects at this level need to be considered in order to answer certain research questions. One example would be the influence of regulatory measures implemented on a macro (ie, national level) such as travel-related control or social distancing measures that critically influence the effectiveness of measures implemented on the school level to prevent and control the spread of severe acute respiratory syndrome coronavirus 2 (SARS-CoV-2).

## Employing a Plurality of Methods That Attend to Complexity

 Applying a complexity science lens and system thinking to implementation science and practice has implications for conceptualizing and analysing context.

 As outlined above, conceptualizing context, implementation and the intervention as separate entities may not produce valuable insights into why some interventions achieve effects and others do not. Indeed, the separation of context and intervention has been perceived as difficult and artificial.^9^ Nevertheless, stakeholders also acknowledge that it is sometimes necessary in order to improve the understanding of how an intervention (ie, event) reconfigured the context at a certain point in time.^9^ Drawing on complexity science, negotiating what constitutes the intervention of interest and what constitutes context can provide valuable insights into domains requiring adaptation and identify mechanisms of how to achieve this change. Also, negotiating the boundaries of a respective system within the research team and together with stakeholders can further the understanding of the systems and its mechanisms.

 In equal measure, researchers need to be reflective and decisive about which domains and levels of context are important and need to be considered in order to describe the system relevant for the question at hand. These decisions should ideally be documented and adequate methods to assess these domains need to be chosen. For example, it might be relevant to assess cultural aspects at a macro (eg, society), meso (eg, organization) and micro (eg, profession) level when assessing the adherence to masks during the coronavirus disease 2019 (COVID-19) pandemic in different settings.

 Assessing these aspects on potentially multiple levels will require a plurality of methods that attend to the complexities within a respective system. A thorough assessment or analysis of context ideally entails a continuous assessment of aspects which are deemed relevant for the intervention of interest. The complexity of the issue at hand is likely to require not only mixing methods, but also the application of methodological pluralism. This can comprise case study research,^14^ participatory research,^6^ ethnographic approaches^15^ but also make use of methods from other disciplines that are more adequate to assess the domains of interest to the intervention at hand (eg, document and media analyses as pursued in communication science; stakeholder analysis as pursued in the political sciences^6^; social network analysis as pursued in the social sciences^6^). In order to account for the dynamic nature of context, researchers should moreover consider a longitudinal rather than a cross-sectional design when implementing an intervention.^11^

## Conclusion

 The conceptualization of context as well as its operationalization in implementation science theory and practice will remain a challenge. Complexity theory and corresponding methodologies might offer some food for thought and guidance for researchers aiming to operationalize context. While these might not be sufficient in fully accounting for complexity, embracing an organic view on the intervention – context dyad can further our understanding of implementation efforts. Along the way, researchers will be required to make decisions about the concept of context, negotiating the boundaries of a system, its key domains and levels requiring a more in-depth assessment. A plurality of methods should be employed to adequately assess this complexity. The findings gained through this route of inquiry will further the understanding of context in the field and its representation in implementation science FMTs and thus contribute to the advance of the field and the re-examination of prevailing paradigms.

## Ethical issues

 Not applicable.

## Competing interests

 Author declares that she has no competing interests.

## Author’s contribution

 LMP is the single author of the paper.
